# Present and Future of the Poor-Law Infirmary

**Published:** 1913-06-28

**Authors:** 


### PRESENT AND FUTURE OF THE POOR-LAW INFIRMARY.

### Bacteriological Research in Infirmaries.

MODERN medical and surgical science, as applied to the treatment of patients in a modern institution and to the cure or alleviation of suffering and disease, has few handmaids so potent for good as bacteriology. This is recognised in every modern hospital, where the bacteriological department is one of the most important and costly of all the departments that the institution possesses. It is becoming more clearly recognised in private practice every day, since the facilities afforded to practitioners by clinical research societies and institutions for the examination of specimens of tissue and fluid are increasing. That being the case, it is only natural that the infirmary should strive to emulate other institutions and give to its workers the same facilities which workers in a modern hospital possess in their investigation and treatment of disease.

The bacteriological laboratory in an infirmary need not be elaborate or costly. As a type of such a working department we may instance the small bacteriological laboratory attached to the London Fever Hospital. This is a small room placed in the mortuary block, and affording accommodation for about three workers. Ordinarily, since the staff of the hospital is limited, it is used only by the resident medical superintendent and his assistant. Two gas-heated incubators are provided, with adequate arrangements for the cultures and for examining specimens under the microscope. It is moderately easy to include in such a laboratory the simple armamentarium which is required for the preliminary examination, chemical, cytological, and microscopical, of morbid fluids and of ordinary body secretions such as urine and fæces. Where such facilities are provided it will usually be found that one or other of the medical staff takes sufficient interest in one department or one branch of investigation to make something of a speciality of it. It is this kind of specialisation that is eminently desirable in the infirmaries. With their vast amount of clinical material—often of a type that lends itself peculiarly to investigation, and often presented in a form for which the hospital investigator seeks in vain—they afford valuable means for investigating certain problems, especially in regard to chronic diseases. A great deal of very excellent work has already been done by infirmary workers who have prosecuted their studies in special laboratories upon material derived from infirmaries, but much more may be done in the future if this necessity for adequate laboratory provision is once fully recognised. The modern infirmary should possess a special clinical, bacteriological, and chemical laboratory, equipped with the ordinary appliances which are required by workers who desire to deal with the ordinary everyday questions of diagnosis that arise in the course of the routine work of the institution. To provide such a laboratory does not mean to embark upon a scheme of costly reconstruction. Nor does it mean the provision of expensive and exceedingly delicate apparatus. A vacant room, now used as a store-room, may often be turned into a good working laboratory with a little trouble and a little expense in fitting it up. An adequate water supply, good lighting, and efficient ventilation are, after all, not so difficult to guarantee in an institution which possesses its own water supply and has a good system of lighting. The initial cost of providing good and efficient microscopes, centrifugal machines, incubators, working tables or slabs, shelving, burners, dishes, bottles, culture media, and other necessities, may at first glance appear to be large, but in reality, taking into consideration the resultant good that comes from such investigation as will be done in a laboratory of this kind, the expense is trifling in comparison with the results obtained and the subsequent profit to the institution.

It is sometimes argued that the work in an infirmary is too strenuous and too exacting to permit any of the workers to "dabble" in the laboratory. This argument falls to the ground when it is admitted that something might, and indeed must, be done to apportion the work more evenly so as to give individual workers a chance to do original research if they feel inclined to do so. Much of the work that the infirmary doctor does to-day is dresser's work; with better nursing and the training of nurses to do the dressings, the medical staff will be in a position to carry out research work, since more time will be afforded to individual workers. Even without such a rearrangement, much may be done by a worker who carefully maps out his day's work, scheduling his duties in such a manner that he gets at least one hour a day free and undisturbed for special investigation; but such investigation should be done on the infirmary premises. The worker who has to go outside to investigate and examine his specimens is sadly handicapped. His time is taken up in going from and coming back to to the infirmary where he works and resides. If provision is made for a laboratory on the premises he will have facilities which he cannot otherwise obtain, and consequently much of his time will be saved. In some good infirmaries the suggestions made above have already been carried out. These institutions are admirably equipped, and are provided with laboratories that are as good for working purposes as those that exist in many hospitals. But the principle which secured the provision of these laboratories has not been universally recognised, and there are still many institutions, large as well as small, which afford the worker no facilities whatever for doing such a simple clinical experiment as a blood count under conditions that safeguard the result from being inaccurate and misleading. The reform in these institutions depends largely, if not entirely, upon the workers themselves. If they would make their wants felt, if they would persistently and judiciously agitate for these facilities, we feel confident that it would be merely a matter of time before their wants were provided for in a manner that is satisfactory to all concerned.

The bogey of cost is one which the authorities are likely enough to raise when an appeal is made, but it is a scarecrow that should have no existence in a modern institution where the authorities appreciate the value of modern medical science and its ancillaries in the treatment of patients. Once it is shown that modernising and improving the means of diagnosis and treatment result in increased efficiency, and therefore inevitably in increased economy, the authorities will be the first to appreciate the interest of the worker that prompted such agitation and secured such provision.

